# Resolving the predator first paradox: Arthropod predator food webs in pioneer sites of glacier forelands

**DOI:** 10.1111/mec.14839

**Published:** 2018-09-07

**Authors:** Daniela Sint, Ruediger Kaufmann, Rebecca Mayer, Michael Traugott

**Affiliations:** ^1^ Department of Ecology University of Innsbruck Innsbruck Austria

**Keywords:** feeding network, global warming, molecular gut‐content analysis, mountain ecology, primary succession, trophic interactions

## Abstract

Primary succession on bare ground surrounded by intact ecosystems is, during its first stages, characterized by predator‐dominated arthropod communities. However, little is known on what prey sustains these predators at the start of succession and which factors drive the structure of these food webs. As prey availability can be extremely patchy and episodic in pioneer stages, trophic networks might be highly variable. Moreover, the importance of allochthonous versus autochthonous food sources for these pioneer predators is mostly unknown. To answer these questions, the gut content of 1,832 arthropod predators, including four species of carabid beetles, two lycosid and several linyphiid spider species caught in early and late pioneer stages of three glacier forelands, was screened molecularly to track intraguild and extraguild trophic interactions among all major prey groups occurring in these systems. Two‐thirds of the 2,310 identified food detections were collembolans and intraguild prey, while one‐third were allochthonous flying insects. Predator identity and not successional stage or valley had by far the strongest impact on the trophic interaction patterns. Still, the variability of prey spectra increased significantly from early to late pioneer stage, as did the niche width of the predators. As such the structure of pioneer arthropod food webs in recently deglaciated Alpine habitats seems to be driven foremost by predator identity while site and early successional effects contribute to a lesser extent to food web variability. Our findings also suggest that in these pioneer sites, predatory arthropods depend less on allochthonous aeolian prey but are mainly sustained by prey of local production.

## 1 INTRODUCTION

Understanding which processes drive primary succession has been a fundamental and long‐standing topic in ecological research, including initial colonization patterns and the development of trophic webs of the colonizer communities (Clements, 1916; Lawton, 1987). In contrast to the classical bottom‐up formation of trophic webs on remote sites such as islands (Lindroth, Andersson, Bödvarsson, & Richter, 1973), it has been recognized that on sites with fully developed ecosystems nearby, the initial communities may include strong heterotrophic components even before the local establishment of vascular plants (Hodkinson, Webb, & Coulson, 2002). This phenomenon has been termed the “predator first paradox” and was described from forelands of retreating glaciers (Hågvar, 2012), volcanic deposits such as lava fields and pyroclastic flows (Ashmole & Ashmole, 1987; Edwards & Sugg, 2005) and sand dunes (Lowrie, 1948). These pioneer sites are highly dynamic habitats, frequently disturbed by abiotic factors such as run‐off water, movement of debris, mudflows and repeated freeze‐thaw cycles (depending on latitude and altitude). Pioneer sites that are exposed to primary succession by ongoing glacier retreat are the best‐studied type of these ecosystems. The common features of their initial invertebrate communities across climatic zones and geographic regions (review by Hågvar, 2012) are generalist predators or scavengers such as spiders, carabid beetles, and harvestmen and microarthropod grazers/decomposers, typically collembolans and mites. Although the suite of taxonomic groups is highly similar across glacier forelands, the community composition, that is, the relative abundance of taxa, can vary significantly even at small geographic scales (Gereben, 1995; Hodkinson, Coulson, & Webb, 2004; Vater & Matthews, 2015).

While species composition and successional development at sites which have recently become ice‐free (1–20 years) are studied quite well, little is known on which prey sustains the predators colonizing these pioneer sites where primary production by plants is either absent or has just started. For arthropod predators residing in these pioneer sites either locally produced, autochthonous prey such as collembolans or allochthonous, external prey brought in by wind or actively flying into the pioneer sites (aeolian input) is available (Hodkinson et al., 2002). Such aeolian prey was found to be an important food source for arthropod predators in pioneer sites in Spitsbergen where high numbers of linyphiid spiders capture aeolian prey in their webs, making it available to the arthropod predator food web (Hodkinson, Coulson, & Harrison, 2001). However, pioneer predator communities in other regions such as the Alps differ in their community composition and include high proportions of larger, nonweb‐building species, mainly carabid beetles and lycosid spiders (Gobbi, de Bernardi, Pelfini, Rossaro, & Brandmayr, 2006; Gobbi et al., 2007; Kaufmann, 2001). For these alpine sites, stable isotope signatures obtained from arthropod predators caught in pioneer sites suggest that locally available decomposer prey, that is, collembolans, is a main dietary component (König, Kaufmann, & Scheu, 2011). Moreover, these data also suggested that intraguild predation occurs at a significance level among these alpine pioneer predators, which are all generalist and therefore capable of feeding on each other. In general, intraguild predation is expected to be high when the availability of extraguild prey is low (Lucas & Rosenheim, 2011; Polis & McCormick, 1987), which can be expected to be a typical situation for pioneer sites in glacier forelands. Indeed, intraguild predation was corroborated to frequently happen by a follow‐up study combining the stable isotope approach with molecular gut‐content analysis (Raso et al., 2014), albeit extraguild aeolian prey was not considered in this work.

Predicting which prey sustains such pioneer predator communities is also hampered by intra‐ and interspecific changes in trophic networks which are expected to be most pronounced in communities which harbour generalist consumers, as these will be most flexible in responding to changes in food availability (Scheu, 2001). This dietary flexibility is of particular relevance for macroarthopod colonizers of the highly disturbed pioneer stages of glacier forelands, where the local food sources, autochthonous microarthropods, can strongly fluctuate in space and time (Hågvar & Ohlson, 2013; Hågvar & Pedersen, 2015; Matthews & Vater, 2015). Likewise, the availability of allochthonous insect prey can be extremely patchy and episodic in these environments (Edwards, 1987; Hawes, 2008; Hodkinson et al., 2001; Ingimarsdóttir, Ripa, Magnúsdóttir, & Hedlund, 2013). For pioneer sites in front of retreating glaciers, all these factors apply, potentially leading to an increased intraspecific variation in food consumption despite high similarities in community composition.

An empirical assessment of such emerging generalist predator food webs, which measures, in a replicated manner, all predators and their actual consumption of the broad range of autochthonous and allochthonous prey, has not been conducted. One of the main reasons for this gap of knowledge is the difficulty in identifying all relevant prey taxa within the gut content of these predators, some of them showing extraoral digestion (spiders) and/or macerating prey before ingestion to render it unidentifiable when examining the gut content by microscopic means (Traugott, Kamenova, Ruess, Seeber, & Plantegenest, 2013). Molecular methods can overcome such methodological hurdles in prey identification and allow to empirically examining which prey is consumed by generalist arthropod predators at pioneer sites. The obtained frequencies of trophic interactions provide a good proxy for interaction strengths (Baker, Buckland, & Sheaves, 2014) and allow for comparisons between different study sites when the same detection systems are applied.

Employing the molecular approach, we here address these knowledge gaps by investigating the diet of over 1,800 individual predators (carabid beetles, lycosid and linyphiid spiders) which were collected in early (0–8 years ice‐free) and later (13–20 years) pioneer stages in three neighbouring glacier valleys in the Austrian Alps. Each individual predator was analysed for the recent consumption of 20 prey taxa using a series of multiplex PCR assays. The development of these assays, specifically designed to study the food choice in these pioneer arthropod communities, was informed by a long‐term research campaign characterizing these systems (e.g., Kaufmann, 2001; Kaufmann, Fuchs, & Gosterxeier, 2002; Kaufmann & Juen, 2001; Kaufmann & Raffl, 2002), allowing to examine the broad potential food spectrum. By detecting more than 2,300 feeding interactions, we investigated the importance of allochthonous flying insects, autochthonous collembolans and intraguild prey for the arthropod predators in early and late pioneer stages. Furthermore, we tested the influence of valley, predator identity and time since deglaciation on the recently established food webs. We posit that these pioneer generalist predators show high intraspecific diet variation or an overall opportunistic, unselective feeding behaviour due to the harsh, food limited conditions. This should result in a high variability of the diet composition within the individual predator taxa due to the fluctuations in the available prey between valleys and pioneer sites, respectively. Consequently, differences in the trophic interactions should be most pronounced between sites (i.e., valleys and early/late pioneer stage, respectively) and less determined by consumer identity. We also predict that within valleys, the anticipated relatively higher food density of extraguild prey in the late compared with early pioneer stages should enable the predators to broaden their prey spectrum.

## 2 MATERIALS AND METHODS

### 2.1 Field sites

Field sampling was conducted in July 2010 on glacier forelands in three valleys in Tyrol, Austria; namely Gaisbergtal (46.835°N, 11.057°E), Rotmoostal (46.824°N, 11.044°E) and Langtal (46.803°N, 11.007°E). This region can be deemed ideal for the present study, as especially the glacier foreland in Rotmoostal has been investigated since many years by scientists of various disciplines such as botany, ecology, geology, limnology, microbiology and zoology. Therefore, the sampling area is very well known and extensive background information is available (e.g., Erschbamer, Niederfriniger Schlag, & Winkler, 2008; Füreder, Schütz, Wallinger, & Burger, 2001; Kaufmann, 2001; Tscherko, Rustemeier, Richter, Wanek, & Kandeler, 2003). The three valleys were chosen as replicates under similar environmental conditions (Supporting Information Appendix S1) to account for variation between investigated arthropod communities. They lie more or less in parallel beside each other and have a glacier at their head. The mountain ridges separating the valleys elevate to approx. 2,700 and 3,300 m a.s.l., and beeline distances between the three glacier forelands are 1.5 and 3.75 km, respectively. Since the last glacial maximum around 1850, the glaciers retreated between 1.5 and 2 km and in 2010 the glacier snouts were at about 2,500 m a.s.l.

**Figure 1:**
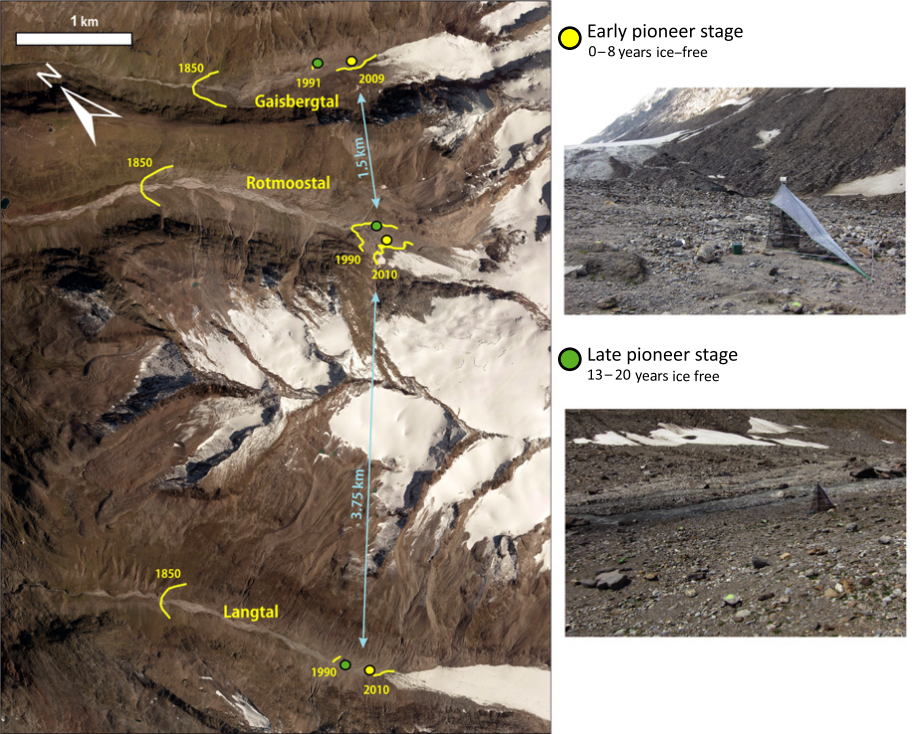
Map of the study sites. Yellow lines represent dated glacial positions. Locations of early (yellow) and late (green) pioneer stages are marked with circles. Pictures to the right provide examples from the habitat at the early and late pioneer stages. Detailed maps of the study sites are available as Supporting Information Appendix S2 [Colour figure can be viewed at wileyonlinelibrary.com]

Per valley, two sampling sites were selected close to the glacier snout: an early pioneer stage covering the area being ice‐free for 8 years or less, and a late pioneer stage covering the area being ice‐free for 13–20 years. The extension of the individual sampling areas was based on annual GPS tracks of the glacier in Rotmoostal and the official measurements of glacial retreat by the Austrian Alpine Association for Langtal and Gaisbergtal. A graphical overview on the study sites is given in Figure 1, and detailed maps of the sampling sites are provided as Supporting Information Appendix S2. While the early pioneer stages are vegetation free on large parts (vegetation cover approx. 2%), first individuals of cushion‐forming plants have had established on the late pioneer stages (vegetation cover approx. 15%). Thus, with only a few plant species present (e.g., *Linaria alpina*,* Saxifraga aizoides*,* S. oppositifolia*), the late pioneer sites represent the initial stage of plant succession (Nagl & Erschbamer, 2010).

On each sampling site, a grid of dry pitfall traps with a spacing of ~10 m was established to collect predatory arthropods, mainly carabid beetles and lycosid spiders. In Rotmoostal, already existing grids (75 and 50 traps in early and late pioneer stage, resp.) were used (Raso et al., 2014), and 50 pitfall traps each were installed in the other two valleys per site. Sampling started shortly after snowmelt in the area and was conducted in parallel in the three valleys between the 12th and 23rd of July 2010. After this date, a sudden drop in temperature accompanied with snowfall occurred on the study sites, ending the main activity phase of the targeted arthropod predators for this year. This was confirmed by the ongoing monitoring of predator activity in Rotmoostal until the 10th of August, where only a total of 245 carabid beetles (100 regurgitates) and 22 lycosid spiders were found in all pitfall traps, compared to 959 carabid beetles (562 regurgitates) and 153 lycosid spiders in the 2 weeks before. Predators were collected alternatingly on the early and late pioneer stages during 24‐hour periods. Traps in the established grids could be quickly inactivated/activated every 24 hr by placing/removing a piece of netting that served as “rope ladder” and allowed animals to escape inactive traps. Due to the reduced surface activity of linyphiid spiders, they are underrepresented in catches from pitfall traps and were additionally hand‐collected by turning stones from five 1 m² plots per day.

Upon collection, predators were individually placed in reaction tubes, kept cool and within approx. 4 hr transported to a nearby field station for further sample processing. Considering this handling and the anticipated detection window of about 2–4 days in these high alpine arthropod predators (Sint, Raso, Kaufmann, & Traugott, 2011), it is highly unlikely that the sampling procedure favoured DNA degradation and had adverse effects on the results of the molecular analysis. See Supporting Information Appendix S7 and Raso et al. (2014) for a more detailed description of field sampling procedures of predators and prey.

### 2.2 Species community

Close to the glacier snout, the predatory arthropod communities of the investigated valleys consist of four species of Carabidae (Coleoptera; *Nebria germari* Heer 1837, *N. jockischii* Sturm 1815, *N. rufescens* (Stroem 1768), and *Oreonebria castanea* (Bonelli 1810)), two species of Lycosidae (Araneae; *Pardosa nigra* (C.L. Koch 1834) and *P. saturatior* Simon 1937), several species of Linyphiidae (Araneae) and one species of Phalangiidae (Opiliones; *Mitopus glacialis* (Heer 1845)) (Kaufmann, 2001). All these arthropod predators have been shown to be residential in those habitats, with all developmental stages being present (Kaufmann, 2001; Kaufmann & Juen, 2001). Despite the relatively close proximity of the early and late pioneer stages (150–280 m centre to centre distance), no considerable movement of individuals between the two pioneer stages was recorded in Rotmoostal with mark–recapture experiments (Baldes, 2009; Brunner, 2011). The lycosid spiders are active during the whole snow‐free period of usually 10–12 weeks in those glacier forelands, and the carabid beetles have their main activity phase in the early season (Kaufmann & Juen, 2001), the time when our sampling was conducted. Adult *M. glacialis,* however, appear only later in the season (late August) and could thus not be included in this study.

The adults of the four species of carabid beetles are easy to distinguish with the naked eye following the descriptions in Freude, Harde, Lohse, and Klausnitzer (2004) such that it was possible to record the species of each individual upon collection. Approximately 60% of the collected adult carabid beetles readily provided regurgitates suitable for gut content analysis (Raso et al., 2014; Waldner & Traugott, 2012) and could be released unharmed after taking samples. As species identification of the spiders is not that straightforward, the lycosid spiders were identified molecularly with a duplex PCR system (Sint, Thurner, Kaufmann, & Traugott, 2015) and the linyphiid spiders handled at family level. All species were found on all six sampling sites except that there was no *P. saturatior* in Langtal and no *N. rufescens* was caught in the early pioneer stages of Gaisbergtal and Rotmoostal, although it was detected as prey in the latter. Supporting Information Table S3 lists the numbers of caught individuals and samples included in the molecular analysis for each taxon and sampling site.

### 2.3 Molecular analysis

To investigate recent meals of the caught arthropod predators, DNA was extracted from beetle regurgitates and whole spiders (50% of the caught individuals) as described in Raso et al. (2014) and consecutively screened with five multiplex PCR systems (Supporting Information Table S4; Sint, Niederklapfer, Kaufmann, & Traugott, 2014; Sint, Raso, & Traugott, 2012; Sint et al., 2015). Thus, we were able to track intraguild predation as well as consumption of Collembola, Hymenoptera, Lepidoptera, Plecoptera, several groups of Diptera (i.e., the subsection Calyptratae, the families Bibionidae, Chironomidae, Phoridae, Sciaridae, Syrphidae, Tipulidae) and *Cinara* sp. (Hemiptera: Aphididae). As Linyphiidae were handled at family level as predators, they were treated the same way as prey. No general primer pair detecting DNA of Linyphiidae is available so far; thus, a multiplex PCR system detecting DNA of five linyphiid species common on the sampling sites was applied (Supporting Information Table S4; Sint et al., 2015) and the results for the individual species combined to “detection of Linyphiidae.”

All PCR products were analysed on QIAxcel (Qiagen, Hilden, Germany), an automated capillary electrophoresis system, with separation method AL320 and the corresponding software Biocalculator 3.2. An expected amplicon length with a signal strength of ≥0.1 relative fluorescent units was counted as a positive detection. The multiplex PCR systems used to screen for intraguild predation included the primer pair targeting the predator itself. As also regurgitates often contain DNA of the consumer (Raso et al., 2014, current study), the applied method cannot discriminate between predator DNA and cannibalism but a signal generated for the predator could be used as internal positive control for the respecitve sample. In case, no PCR product was amplified with any of the multiplex PCR systems, the sample was retested with universal primers. If a sample failed to amplify with those as well, it was excluded from the analysis. In total, this was the case for 30 regurgitates (Supporting Information Table S5), leaving 1,832 predatory arthropods (Supporting Information Table S3) from three different glacier forelands which were analysed for 20 different prey taxa.

### 2.4 Statistical analysis

#### 2.4.1 Predator diet

For each combination of predator by valley by pioneer stage (7 by 3 by 2), the proportion of predators testing positive for the 20 prey taxa was determined (data given in Supporting Information Data S6). These were used for ordination of prey compositions and multivariate tests. Groups with less than 10 predator individuals were excluded from these analyses (no sufficiently reliable proportion estimates possible), and the nondetectable cannibalistic interactions were treated as missing values. For some analyses and graphs, the prey taxa were combined into larger groups (e.g., carabids, lycosids, brachyceran and nematoceran Diptera) to attain sufficient numbers for statistics and for the sake of simplicity. PRIMER 7 with the PERMANOVA+ package (PRIMER‐E Ltd., Lutton, UK) was used for principal coordinates ordination (PCO), resemblance‐based permutation ANOVA (PERMANOVA) and testing differences in variability (PERMDISP) (Anderson, Gorley, & Clarke, 2008). 9,999 permutations were used for all tests.

Prey spectra, that is, the detection frequency of prey taxa found within individual predator species, were analysed by generalized linear models (GLM) in R 3.3.2 (R Core Team 2016). The binomial or, in case of overdispersion (residual deviance larger than the degrees of freedom), the quasibinomial family on prey detection counts from all three valleys (grouped prey taxa as above; very rare prey taxa with <5 detections overall excluded) was used. Models to assess differences between the early and late pioneer stages tested the interactions *prey type by stage* after accounting for all significant main and interaction effects of prey type and valley.

#### 2.4.2 Food webs

Following a frequency of occurrence approach to determine the interaction strength between individual predator–prey–pairs (Baker et al., 2014), the molecular presence–absence data from each individual was transformed into the relative diet composition for the whole taxon (i.e., proportion each prey type contributed to the total detections in this predator taxon) and plotted with Food Web Designer 3.0 (Sint & Traugott, 2016). In addition, H_2_ (Blüthgen, Menzel, & Blüthgen, 2006), which is relatively insensitive to sample size, was calculated as a quantitative index for network‐level specialization (based on predation frequencies). For single predator taxa, Hill's N1 (Hill, 1973) was used to characterize niche width.

## 3 RESULTS

### 3.1 Community composition

Although the suite of present taxa was nearly identical between the six sampling sites (Supporting Information Data S7), the absolute and relative abundances of both predators and the potential prey diverged: For the predator community, a stronger difference was found between valleys and a weaker difference between the successional stages, while the opposite was true for the prey community. Due to a strong increase in flying insect abundances and a reduction in collembolans on the late pioneer stage, successional stages had a larger influence on the composition of the prey community than the affiliation to one of the three valleys (details and ordination graphs in Supporting Information Appendix S7).

### 3.2 Trophic interaction network

Prey spectra and interaction strength were analysed for differences between predators, the three valleys and the changes from the early to the late pioneer stage. Contrary to the expectation, that differences in trophic interactions would be highest between sites, the three‐way PERMANOVA based on Bray–Curtis similarities revealed that predator identity was by far the strongest factor for trophic interactions (pseudo‐*F*_6,32_ = 3.98, *p* = 0.0001, explained SS 38.6%). The identity of the valley showed the second largest effect (pseudo‐*F*_2,32_ = 4.52, *p* = 0.0001, explained SS 14.6%), while the time since deglaciation (early vs. late pioneer stage) was the weakest, but still highly significant factor (pseudo‐*F*_1,32_ = 2.84, *p* = 0.0096, explained SS 4.6%). Interactions between the analysed factors were not significant and were therefore dropped from the PERMANOVA design.

**Figure 2:**
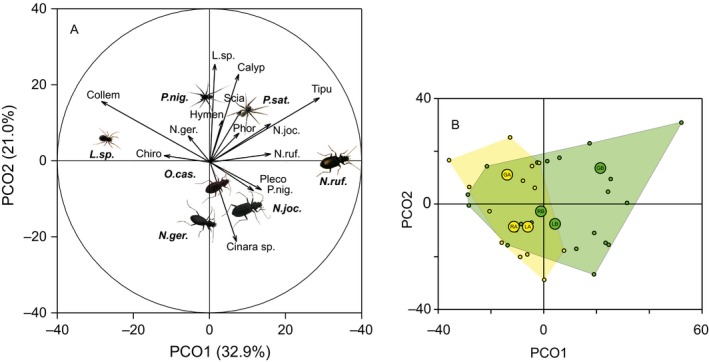
Ordination of prey composition for predators by valley and pioneer stage. Principal coordinates analysis (PCO) on Bray–Curtis similarity. Panel (A): predators (pictures at centroids with bold labels) and prey (arrows indicating correlations with axis referenced to the unit circle drawn; only taxa with |*r*| > 0.3 shown). Panel (B): Centroids for the three valleys Gaisbergtal (G), Rotmoostal (R) and Langtal (L) are shown for early (GA, RA, LA; yellow) and late (GB, RB, LB; green) pioneer stage. Position of the single data points in the same ordination space as panel (A) together with the envelopes for the two pioneer stages [Colour figure can be viewed at wileyonlinelibrary.com]

The pattern of these differences is shown by the ordination in Figure 2 (PCO on Bray–Curtis similarity) with two strong axes, representing 32.9% and 21.0% of the variation. Axis PCO1 separated the predators Linyphiidae and the carabid *N. rufescens* (nearly absent in the early stage) and was most strongly associated with high consumption of Collembola in the early stage versus high consumption of Tipulidae in the late pioneer stage (Figure 2A,B). Axis PCO2 separated the two species of wolf spiders (*Pardosa*) from the other three carabid beetles towards higher predation on Linyphiidae and calyptrate flies (Muscidae and Anthomyiidae in particular) and an opposing, but weaker correlation for the aphid *Cinara* sp. There was also a slight separation of one valley (Gaisbergtal) from the other two along axis PCO2 (Figure 2B).

The variability of prey spectra increased significantly with successional development, as the data points of the predators from the late pioneer stage covered an extended range in the ordination space compared to those examined from the early pioneer stage (Figure 2B; variability difference between early and late pioneer stages: PERMDISP, *F*_1,31_ = 6.61, *p* = 0.0112). Similarly, there was a weak trend of a general increase in the predators’ niche widths (Hill's N1) from the early to the late pioneer stage (PERMANOVA, stage effect pseudo‐*F*_1,23_ = 3.338, *p* = 0.083).

Along the same line, the generality index H_2_ increased uniformly in all three valleys from the early (4.62–4.95) to the late pioneer stage (5.04–5.46), with Rotmoostal showing the highest values among the glacier forelands.

### 3.3 Diet of predators

Up to eight different prey taxa were molecularly detected in a single predator individual. The mean number of prey taxa detected per individual was lowest in linyphiid spiders (0.87), intermediate in carabid beetles (1.00–1.64) and highest in lycosid spiders (2.01–2.02).

Niche width (Hill's N1), based on all 20 prey taxa, was significantly lower in the Linyphiidae (2.6) than in the other predators (4.7–7.0 with the highest values in *Pardosa*) (PERMANOVA with factors prey, valley, pioneer stage on Euclidean distances: predator effect pseudo‐*F*_6,23_ = 4.705, *p* = 0.003, pairwise comparisons of Linyphiidae *p* < 0.02 against each of the other predators).

**Figure 3:**
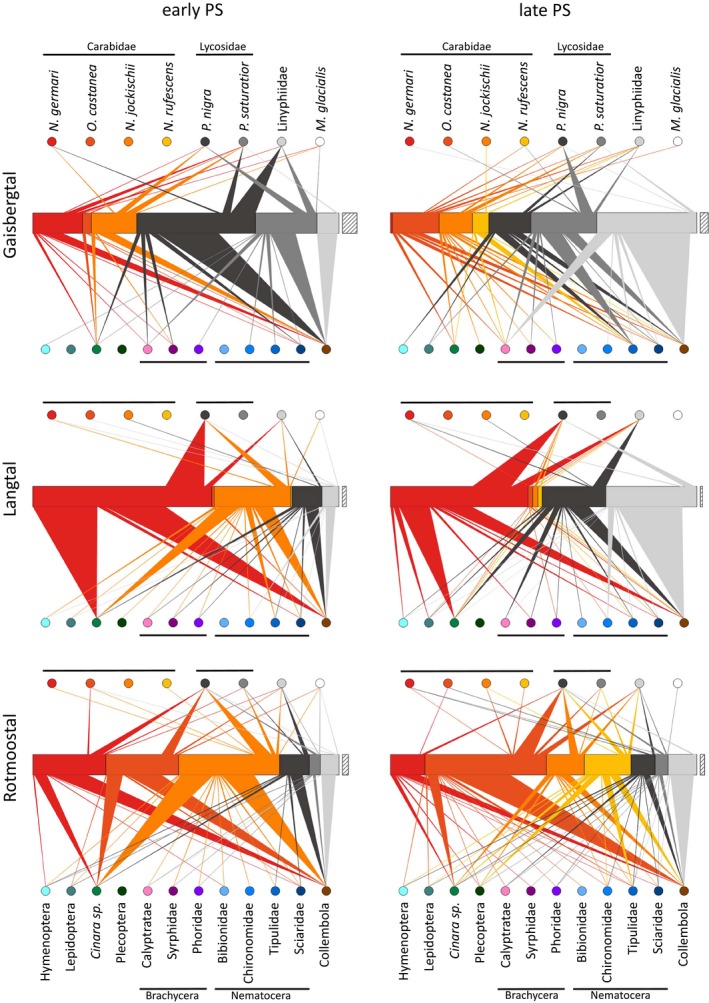
Semiquantitative food webs on early and late pioneer stages (PS) in three glacier forelands. The central bars show the number of predators caught per taxon during the sampling period (12–23 July 2010); the offset bar indicates the width of 10 individuals each. Order and colour of the investigated predator taxa correspond to the labelled circles above the bar. Triangles represent the observed trophic links of intraguild (upwards) and extraguild (downwards) predation. The width of the triangles indicates the percentage each prey taxon contributed to the relative diet composition of the predator (as obtained by molecular analysis of 1,832 predators). As the applied molecular method to track consumption cannot discriminate between predator DNA and cannibalism, no such links are present [Colour figure can be viewed at wileyonlinelibrary.com]

**Figure 4:**
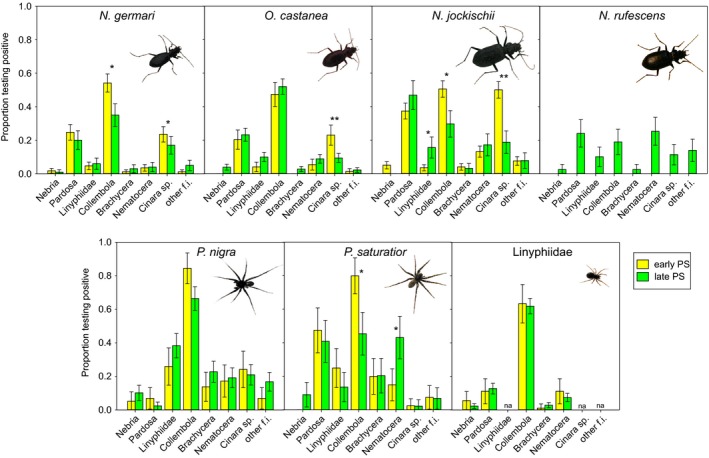
Prey detection rates of the four carabid and three arachnid predators. Combined data from all valleys are shown for early (yellow) and late (green) pioneer stages (with standard errors from GLM estimates). Significant differences between early and late pioneer stage are marked (* *p* < 0.05, ** *p* < 0.01). *N. rufescens* was present only in the late pioneer stage. Prey taxa sorted according to intraguild, autochthonous, allochthonous food sources and combined into groups: Nebria (four carabid species), Pardosa (two species), Nematocera (Bibionidae, Chironomidae, Sciaridae, Tipulidae), Brachycera (Calyptratae (predominantly Muscidae and Anthomyiidae), Phoridae, Syrphidae), and other flying insects (other f.i.: Hymenoptera, Lepidoptera, Plecoptera) [Colour figure can be viewed at wileyonlinelibrary.com]

The molecular data showed that all the predators described above are clearly generalists both in extraguild and in intraguild predation as all tested prey taxa were found in their guts, and they shared many common diet features (Figures 3 and 4). Collembola was overall the most frequently detected prey taxon (44% of detections), followed by DNA of lycosid spiders (16%), but also the various groups of flying insects accounted in total for 30% of the 2,310 detected predation events. Intraguild predation events were rather asymmetrical, as carabid beetles were eaten less frequently than lycosid spiders. Among the flying insects, the aphids *Cinara* sp., which occurred in high numbers episodically and locally and were then found more or less immobilized on the ground, were the most often consumed prey taxon. A similar number of predators tested positive for aphids as for any nematoceran DNA, albeit the consumption of the latter was much more balanced between sites (Figure 3).

Apart from this generalism, there were also some distinct dietary differences between the predators: Among the carabid beetles, there was a markedly lower predation on Collembola by *N. rufescens* (19% vs. 30%–66% of individuals tested positive, present only on late pioneer stage); otherwise, the diets were similar apart from a high variability in their consumption of flying insects. The two species of wolf spiders within the genus *Pardosa* differed from the carabids in their higher predation frequencies on Linyphiidae (14%–38% vs. 4%–16%) and on Brachycera (14%–19% vs. 0%–4%). Remarkably, *Pardosa saturatior* preyed heavily on *P*. *nigra* while the opposite was rarely observed (44% vs. 5%). The small Linyphiidae depended mostly on collembolans (63%) with other prey being consumed only at low rates (Figure 4).

### 3.4 Diet shifts from early to late pioneer stage

Despite the higher variability in the prey spectra of the predators collected on the late pioneer stages (Figure 2B), no clear shift in the diet regarding the use of the intraguild prey, allochthonous flying insects and autochthonous prey component (collembolans) was observed. Overall, the contributions of the different components to the diet of the predators remained roughly constant with approximately 30% allochthonous food (i.e., flying insects) and 70% autochthonous prey, comprising 20%–30% intraguild prey and 40%–50% collembolans. However, on the level of single predators, some specific diet changes from the early to the late pioneer stages were detected (GLMs on the prey taxa grouped as in Figure 4). Most notably, Collembola were less used as prey in the late pioneer stage, where they were also much less abundant, by *N. germari* (*p* = 0.014), *N. jockischii* (*p* = 0.047) and *P. saturatior* (*p* = 0.011) while they remained remarkably constant in *O. castanea* and the Linyphiidae. The aphid *Cinara,* with a highly variable occurrence, was more preyed upon in the early pioneer stage by the carabids *N. germari* (*p* = 0.025), *O. castanea* (*p* = 0.005) and *N. jockischii* (*p* = 0.005). Only the wolf spider *P. saturatior* exploited the higher availability of Nematocera in the late pioneer stage (*p* = 0.021). More detailed information on abundances of prey and differences in the prey spectra between the three valleys is provided as Supporting Information Appendices S7 and S8, respectively.

## 4 DISCUSSION

The present study provides a comprehensive investigation of trophic interactions on recently deglaciated land by sampling over 1,800 individuals of all taxa of arthropod predators present, covering the full range of their main activity phase and considering all relevant prey taxa representing the three potential components of their prey spectrum (allochthonous flying insects, autochthonous collembolans and intraguild prey). To our knowledge, this is so far the most complete arthropod food web being disentangled molecularly. Furthermore, by including three different valleys as replicates, the results go beyond a single system where observations might be strongly influenced by site‐specific factors and thus allow to elucidate more general patterns in these pioneer arthropod predator food webs.

Our data confirmed that all investigated predators are generalist consumers, making use of all 20 tested prey taxa. Thus, all three potential food components proved to be important and contributed—based on the detection frequency of the prey DNA—roughly one‐third each to the relative diet composition of the predators. Furthermore, only minor shifts in the overall use of the components were observed between valleys or early and late pioneer stages, respectively. Contrary to our expectations, the feeding interactions were strongly influenced by predator identity and were similar between the different valleys and successional stages, respectively, despite the differences which were observed in the available prey. However, especially the larger carabid beetles and lycosid spiders did make use of the higher availability of flying insects on the late pioneer stages, leading to a significantly higher variability in the prey spectra of the predators and an increase in trophic niche width in late compared with early pioneer stages.

Despite the pronounced differences in community compositions, where the predators were influenced by the affiliation to the valley and the prey community by time since deglaciation, their trophic interaction patterns remained remarkably constant. Predator identity had by far the highest influence on the diet, and effects of study site (i.e., successional stage and valley) were much smaller, although still statistically significant. This was an unexpected finding, as the available food sources varied significantly between sites. Given that all investigated predators are generalist consumers (Symondson, Sunderland, & Greenstone, 2002), a higher variability in their diet between sites would be expected, according to the observed differences in prey availability (Araujo, Bolnick, & Layman, 2011). Still, the fact that the episodically occurring peaks of specific prey such as *Cinara* sp. aphids were reflected in high detection rates indicates that opportunistic feeding, by making use of randomly occurring and easy‐to‐catch prey, may play an important role for predatory arthropods dwelling in pioneer sites. Beyond that, however, the present findings emphasize significantly different prey choice between predators, meaning that within their generalist feeding behaviour, each predator species has its specific composition of taxa it is preferably preying upon. Compared to the strong impact of predator identity, the observed increase in their dietary diversity from early to late pioneer stage was minor, although it was shown earlier based on stable isotope data that considerable shifts do occur on later successional stages being ice‐free for over 50 years (König et al., 2011). This pattern was constant across the three investigated glacier valleys, suggesting that each predator species on the pioneer stages relies on its specific subset of the available prey diversity and occupies its specific trophic niche.

Apart from these aspects, factors such as microhabitat diversity and environmental stability are known to impact the realization of trophic niches (Araujo et al., 2011). Thus, the high microhabitat diversity known to be characteristic for early successional stages in glacier forelands seems to facilitate trophic differentiation, while intraspecific differences in prey preferences as described by Start and Gilbert (2017) seem to play a minor role in these pioneer food webs.

Combining the findings from the current study with knowledge gained earlier (e.g., Ingimarsdottir, Michelsen, Ripa, & Hedlund, 2014; König et al., 2011; Raso et al., 2014), a pattern emerges on how predator communities on pioneer sites in front of receding glaciers are most likely sustained. First, local productivity is exploited and seems to be the main source of nutrients. While in our case, the local productivity entered the food web nearly exclusively via consumption of collembolans, other mesofauna such as mites, which were hardly present at the investigated sites, might also be of importance (Hågvar, Solhøy, & Mong, 2009). The same holds true for nematodes or protozoans, which were so far not considered in this context. By including the mesofauna into the food webs, it also needs to be investigated in more detail, what sustains them. So far an “invisible” autotrophic productivity by algal biofilm and mosses as well as wind brought organic material entering via a decomposer food web, and nutrients released out of the glacier have been identified (Hågvar, Ohlson, & Flø, 2017).

In cases where local clear water bodies such as spring‐fed streams, lakes or ponds are abundant, insect emergence, mostly chironomids, can be high and thus an important food source too, as has been reported from Scandinavian glacier forelands (e.g., Hågvar, Ohlson, & Brittain, 2016; Hågvar & Pedersen, 2015). However, due to the different topology in the Alps, where extended plains in front of retreating glaciers are rather uncommon, such clear water bodies are less abundant. As the aquatic production is in general reduced in glacier‐fed streams due to harsh environmental conditions such as constantly low water temperatures or the high turbidity during the melting period (Friberg, Milner, Svendsen, Lindegaard, & Larsen, 2001; Füreder et al., 2001; Lods‐Crozet, Lencioni, Brittain, Marziali, & Rossaro, 2007), the overall aquatic production is lower on these sites with few clear water bodies. Mean chironomid catches in water filled traps placed at the soil surface during fieldwork in the three Alpine valleys (Supporting Information Table S7) reached only 17.4% (early PS) and 1.8% (late PS) of the catches found in the Arctic on sites of similar age (Hodkinson et al., 2001). Accordingly, also chironomid consumption frequency was much lower in the present study compared with findings obtained by morphological gut content analysis of predators in Scandinavian glacier forelands (Hågvar and Pedersen (2015).

For Arctic glacier foreland communities, it has also been proposed that a considerable amount of flying insects is caught by web‐building linyphiid spiders which have been reported to cover large areas with their webs (Hodkinson et al., 2001). However, such extensive spider webs were so far not observed in Alpine glacier forelands. This might be mainly due to the fact that other than in Spitsbergen, where just the small linyphiid spiders occur on the pioneer sites (Hodkinson et al., 2004), large, actively hunting lycosid spiders are common in these habitats in the Alps (Gobbi, Fontaneto, & De Bernardi, 2006; Gobbi, de Bernardi, et al., 2006; Kaufmann, 2001). Our data demonstrated that the lycosids do intensively prey on the smaller linyphiids, eventually leading to a behavioural change in the latter to avoid encountering lycosids. This conforms well to the fact that in the investigated areas, most linyphiid spiders were found beneath stones and did not build webs in the open landscape. Consequently, they were not able to catch and consume significant amounts of flying insects but were mainly preying on surface‐active collembolans. The latter is supported by the high detection rates of collembolan DNA within the linyphiid spiders examined in this study.

Nevertheless, flying insects were an important food source for the whole predator community and accounted in total for about 30% of all prey detections in the present study. The high share of flying insects was not surprising, as allochthonous insects are typically available on recently deglaciated terrain. In early studies on pioneer predator communities, they were even deemed as the most likely food source for arthropod predators at pioneer sites (Hodkinson et al., 2001, 2002; Kaufmann, 2001). Our data do not support this notion entirely but show that flying insects are utilized opportunistically and constitute the contingent part of the food web. For example, the locally restricted mass occurrence of coniferous aphids (*Cinara* sp.) on some sites leads to increased prey detection rates for this taxon, even in the carabid beetles, which otherwise consumed flying insects to a lesser extent.

As the third type of food web interactions, we found intraguild predation to be common in these pioneer predator communities, confirming earlier findings by Raso et al. (2014). As would be expected, the small Linyphiidae were frequently preyed upon especially by lycosids, but also the lycosids were in turn a common prey for the carabid beetles, which served hardly as prey for other predators themselves. Whether this imbalance in intraguild predation among lycosids and carabids is due to the hard exoskeleton of the beetles or the divergent circadian rhythmic (diurnal spiders vs. mainly nocturnal beetles) cannot be answered from the present data.

Considering all the three main food sources (local production, aeolian input, intraguild predation) for arthropod predators on pioneer stages, the present study clearly indicates that the importance of allochthonous input might have been overestimated in earlier studies (e.g., Hawes, 2008; Hodkinson et al., 2001). In fact, up to 70% of the feeding interactions were rooted in local production (collembolans and intraguild predation), resulting in food chains consisting of up to four links based on consumption of collembolans and subsequent intraguild predation. Despite the fact that higher trophic level prey can have detrimental effects on growth and reproduction (Wilder, Norris, Lee, Raubenheimer, & Simpson, 2013), the current pioneer predator food webs had a strong intraguild component including long intraguild food chains. This could be a reason why intraguild predation was not significantly higher at the early compared with the late pioneer sites, as it already is at a level where the predators cannot increase it further to avoid negative nutritional effects. Additionally, this might explain why even the largest predators (*N. jockischii* and *P. saturatior*) frequently consumed the very small collembolans on the early pioneer stages, as this lower trophic level prey is available in higher numbers and might improve their nutrient supply. On the late pioneer stages, however, when the availability of alternative low trophic level prey increases in the form of allochthonous flying insects, these larger prey types are preferred by the large predators. The smaller ones (linyphiid spiders, *N. germari*,* O. castanea*) show, on the other hand, a constantly high predation rate on collembolans, although this prey type was significantly less abundant on the late compared to the early pioneer sites. Furthermore, the increase in potential food sources and the shift from collembolans to flying insects, especially in the spiders and large carabid beetles, should lead to a reduction in niche overlap between the predators and therefore relax food competition on the late pioneer stages.

## 5 CONCLUSION

Contrary to our expectations that food webs in pioneer communities would be strongly influenced by local prey availability, leading to random predation patterns for individual species on different sites (time since deglaciation or valley), predator identity had by far the strongest influence on the observed trophic interactions, despite marked differences in predator and prey community composition. As such, the observed feeding interactions stayed remarkably constant, and even the small dietary shifts between early and late pioneer stages were quite similar for individual predator species. We found that arthropod food webs on pioneer stages are less influenced by changes in prey availability, and thus, the predictability is higher than initially expected. Furthermore, the predators seem to be largely sustained by local production, while allochthonous food sources in the form of aeolian input (flying insects) are consumed less frequently than suggested by previous work. This resolves the predator first paradox, as even in the very first stages of primary succession a considerable amount of local production (collembolans) serves as an important food source for the arthropod predators. A logical next step would now be to investigate the food sources of collembolans in more detail as it is not clear how important allochthonous input (detritus) is for these springtails compared to local production by algae and microbes.

## AUTHOR CONTRIBUTIONS

The study was designed, the fieldwork was performed and the manuscript was written by D.S., R.K. and M.T. The data were analysed by D.S. and R.K. Samples were molecularly analysed by R.M. and D.S. The manuscript was commented by R.M.

## Data Availability

Data sets for the description of the predator and prey community as well as their trophic interactions are provided as Supporting Information Appendices S3, S6 and S7.
