# Supplementary material for: Resolving the predator first paradox: Arthropod predator food webs in pioneer sites of glacier forelands
Source: Mol Ecol. 2018 Sep 7;28(2):336–47. doi: 10.1111/mec.14839 (PMC6378689; doi:10.1111/mec.14839)
Supplement: Supplementary file 1 [file MEC-28-336-s001.pdf]

MOLECULAR ECOLOGY

Supplemental Information for:

Resolving the predator first paradox: arthropod predator food webs in pioneer sites of glacier forelands

Sint D., Kaufmann R., Mayer R., Traugott M.

Daniela.Sint@uibk.ac.at, Ruediger.Kaufmann@uibk.ac.at, Michael.Traugott@uibk.ac.at

Table of Contents:

**S1 – Environmental conditions**	Page 2
**S2 – Maps of sampling sites**	Page 3
**S3 – Collected & analysed predators**	Page 6
**S4 – Overview of multiplex systems**	Page 7
**S5 – False-negative samples**	Page 8
**S6 – Prey detection rates**	Page 8
**S7 – Community composition**	Page 9
**S8 – Prey spectrum in the 3 valleys**	Page 11

MOLECULAR ECOLOGY

S1: Environmental conditions

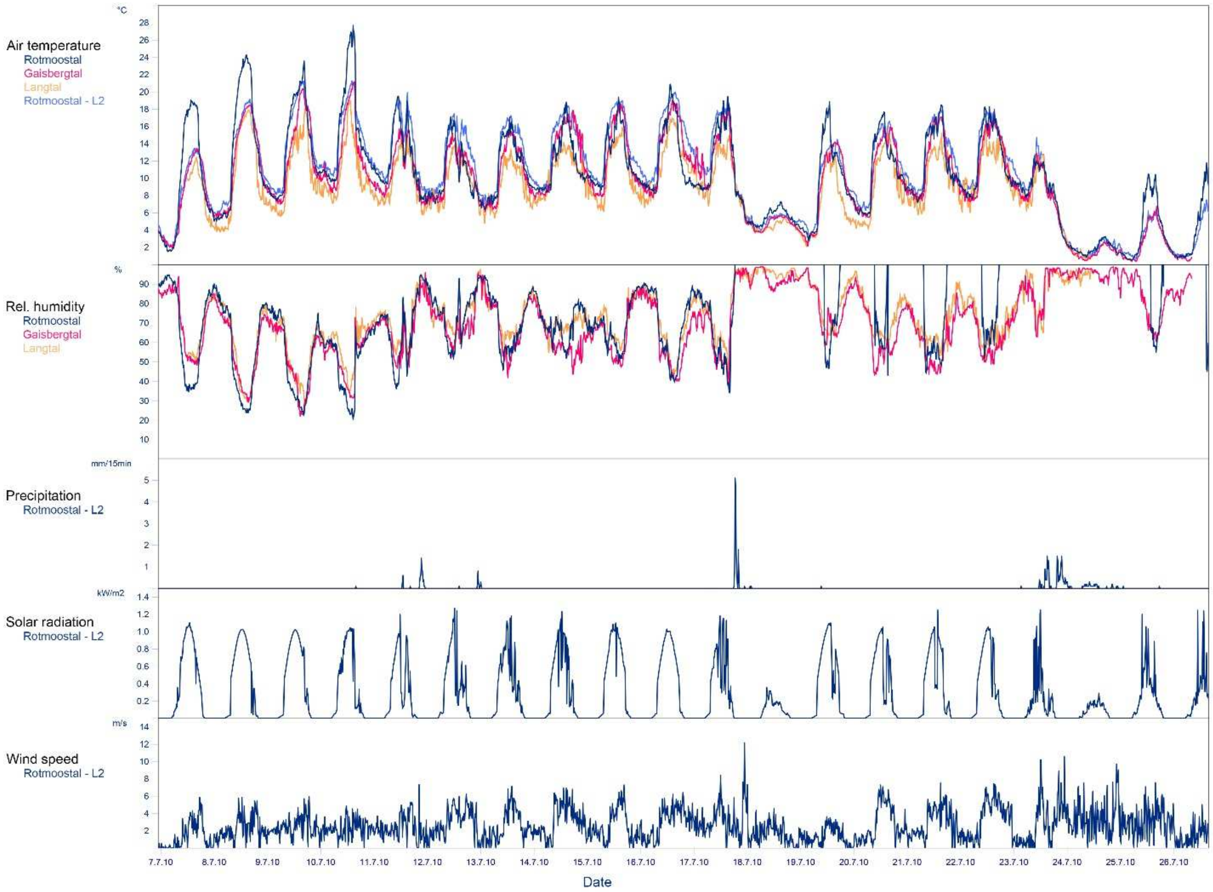

Figure S1: Environmental conditions in the three glacier forelands. Air temperature [°C] and relative humidity [%] were recorded in all three valleys (Rotmoostal, Gaisbergtal, Langtal) in the space between the two sampling sites. In Rotmoostal an additional data logger (Rotmoostal – L2) was operated recording air temperature [°C], precipitation [mm/15min], solar radiation [kW/m²] and wind speed [m/s]. Please note that data has not been validated and cleaned but is displayed as recorded (e.g. radiation error of one temperature logger during the first few days or partial malfunction of the humidity sensor in Rotmoostal following the precipitation on 18th July 2010).

MOLECULAR ECOLOGY

S2: Maps of sampling sites

Yellow lines represent dated ice positions, yellow dots indicate the grid of dry pitfall traps and red dots mark positions of traps for flying insects (one grey bowl and one malaise trap per site)

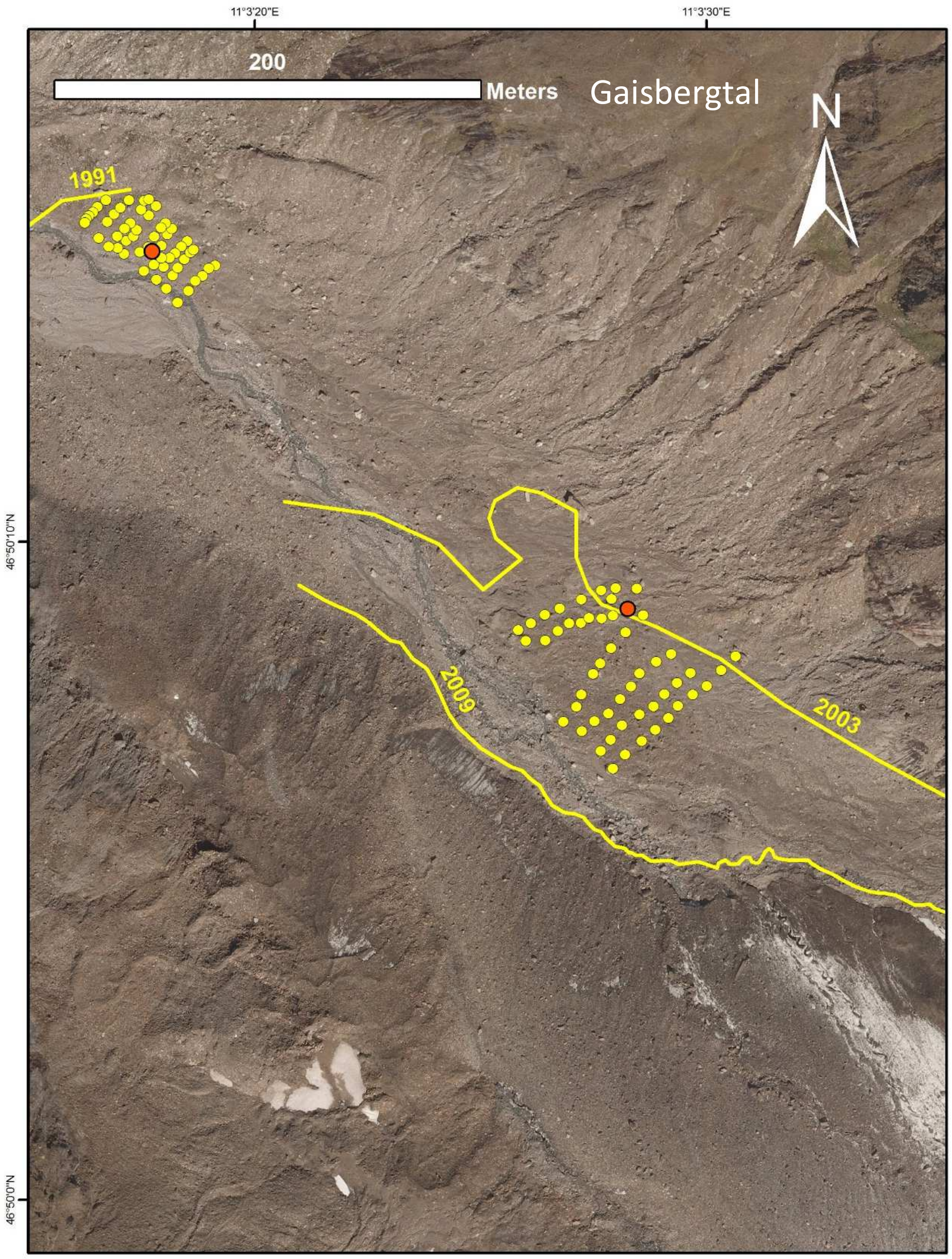

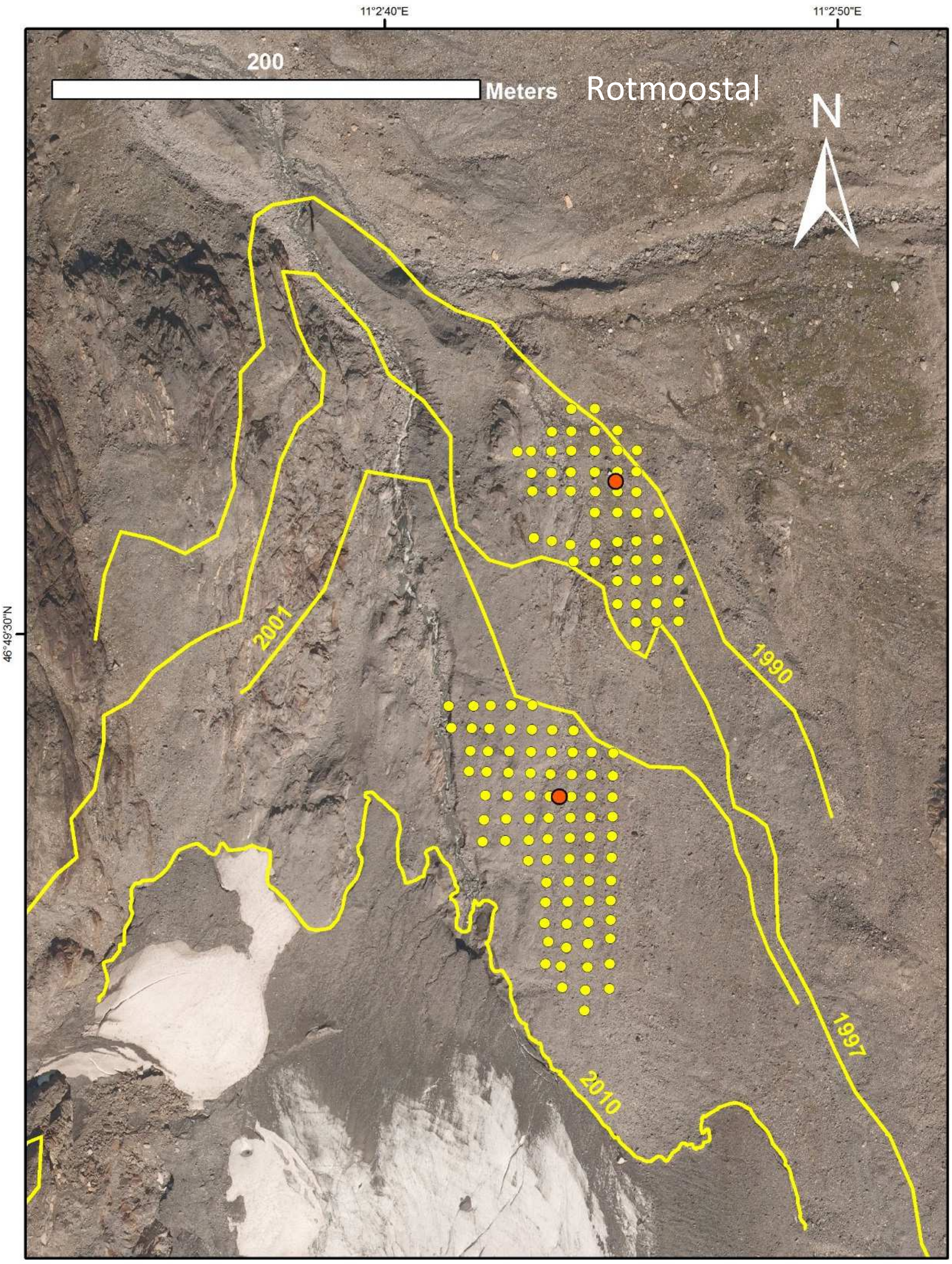
11°2'40"E
11°2'50"E
46°49'30"N
200
Meters
Rotmoostal
N
2001
1990
1997
2010

MOLECULAR ECOLOGY

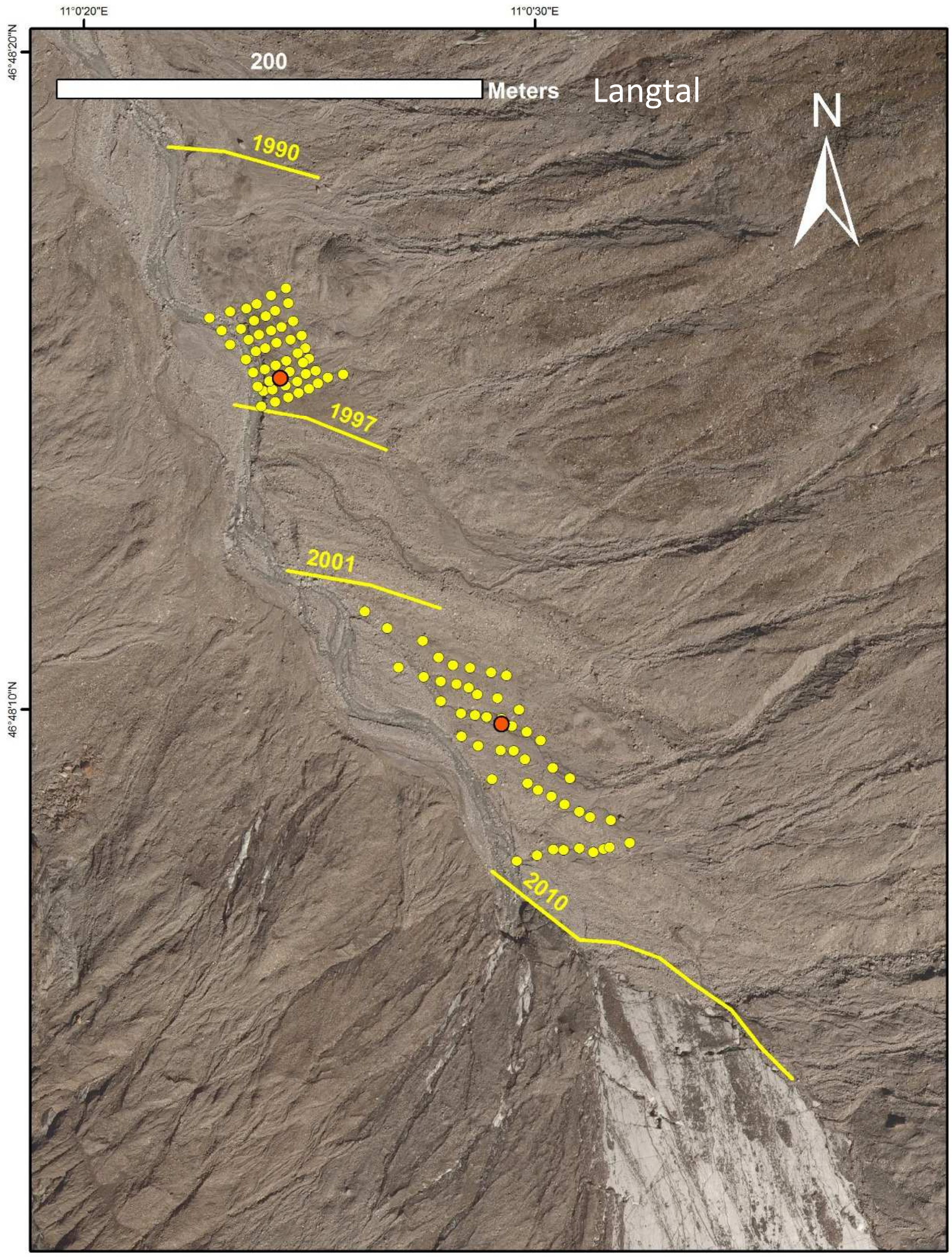

MOLECULAR ECOLOGY

S3: Collected and analysed predators

Table S3: Number of caught (A) and analysed (B) individuals per taxon from the early and late pioneer stage (PS) respectively in each of the three investigated glacier forelands. Note: As the two lycosid spiders could not be identified in the field upon collection their catch-numbers were reconstructed from the ratio found in the analysed samples from each site.

A)

Predator	Total N	Gaisbergtal		Rotmoostal		Langtal	
		**early PS**	**late PS**	**early PS**	**late PS**	**early PS**	**late PS**
Carabidae							
Nebria germari	1267	34	2	125	77	454	575
Nebria jockischii	545	31	41	173	85	194	21
Nebria rufescens	147	2	20	0	104	3	18
Oreonebria castanea	485	6	58	124	271	6	20
Lycosidae							
Pardosa nigra	541	39	52	51	54	78	267
Pardosa saturatior	213	84	81	19	29	0	0
Linyphiidae	657	15	123	32	64	43	380
**Total**	3855	211	377	524	684	778	1281

B)

Predator	Total N	Gaisbergtal		Rotmoostal		Langtal	
		**early PS**	**late PS**	**early PS**	**late PS**	**early PS**	**late PS**
Carabidae							
Nebria germari	270	27	1	99	49	44	50
Nebria jockischii	262	18	20	97	33	83	11
Nebria rufescens	79	0	13	0	52	0	14
Oreonebria castanea	255	3	24	69	145	2	12
Lycosidae							
Pardosa nigra	225	16	22	16	19	26	126
Pardosa saturatior	84	34	34	6	10	0	0
Linyphiidae	657	15	123	32	64	43	380
**Total**	1832	113	237	319	372	198	593

MOLECULAR ECOLOGY

S4: Overview of multiplex systems

Table S4: Overview of the applied multiplex systems. Given are multiplex names, targeted taxa with the respective primer combination, resulting amplicon size and the original publication.

Multiplex	Target	Primer	Primer sequence (5'-3')	amplicon size (bp)	citation
intraguild predation	*Pardosa* spp.	Pard-sp-S238	CTGTTTATCCTCCTTTAGCATCTAC	86	Sint et al. 2012
		Pard-sp-A239	AGCCCCAGCTAAATGAAGAG		
	Nebria rufescens	Neb-ruf-S249	TCAGTCGGAATTACTGCATTAC	107	
		Neb-ruf-A250	GGGTCAAAGAAAGTTGTATTTAAG		
	Oreonebria castanea	Ore-cas-S240	CTCTGTTGACTTAGCTATTTTCAGA	129	
		Ore-cas-A241	AATAAAGGTATTCGATCAAAGGA		
	Mitopus glacialis	Mit-gla-S243	TATACCCCCCTCTATCAAGAAAT	144	
		Mit-gla-A244	TACCTTGTGTTCGTATGTTGATG		
	Nebria jockischii	Neb-joc-S242	GTGAACAGTTTACCCTCCACTG	167	
		Neb-joc-A243	TTCGGTCAAAAGTTATACCAATT		
	Nebria germari	Neb-ger-S241	CGAATGAATAATATAAGATTTTGACTT	198	
		Neb-ger-A242	AGCCCCTAAAATTGAAGAAATA		
	Collembola	Col3F	GGACGATYTTRTTRGTTCGT	228	
		Col-gen-A246	TTTCACCTCTAACGTCGCAG		
Pardosa DUP	*Pardosa nigra*	Par-nig-S258	ATTACCTCCTTCTTTATTTTTATTG	118	Sint et al. 2015
		Par-nig-A257	TATAGAATTTCCTATATGACCAACC		
	Pardosa saturatior	Par-sat-S257	TTGGACATATAGGAAGTTCAATG	202	
		Par-sat-A256	CTAAAACAGGTAAAGAAAGCAAC		
Linyphiidae LIN	*Diplocephalus helleri*	Dip-hel-S278	CCTCCTTCTTTGTTCTTACTATTTG	151	Sint et al. 2015
		Dip-hel-A280	AAGSCCCAGCCAAGTGC		
	Erigone tirolensis	Eri-tir-S281	GGAGCTTGGGCTGCTATAGTA	186	
		Eri-tir-A283	AGGRACTAATCAGTTACCAAAYCCT		
	Janetschekia monodon	Jan-mon-S282	GATATTAGGAGCTCCTGATATAGCC	240	
		Jan-mon-A284	ATAAAATTAATGGCTCCCATAATC		
	Meioneta nigripes	Mei-nig-S287	TCAGATATAGCGTTTCCTCGTATG	264	
		Mei-nig-A288	AGTTATACCATAGCCACGTATATTTAG		
	Entelecara media	Ent-med-S279	GAGYTAGGTCAAGTTGGAAGCC	298	
		Ent-med-A292	ATGCCCCTCTAACGAAGAC		
flying insects FLY-1	Phoridae	Pho-gen-S265	TTCTTTCGGGGATCGTTGAC	82	Sint et al. 2014
		Pho-gen-A266	GACATTTGAAAGATCTGTCGTCG		
	Plecoptera	Ple-gen-S268	TATGGTTCCTTAGATAATACACCA	117	
		Ple-gen-A269	GGTTTTGATCTAATAAAAGCGT	(117-118)	
	Tipulidae	Tip-gen-S267	GCATGTCTAAGTACACACTCTCG	159	
		Tip-gen-A268	ATAAAAGCACACGTTCCTTG		
	Sciaridae	Sci-gen-S266	AGAAACCGGTAAAATGGGT	187	
		Sci-gen-A267	AACCAAGGTAATCCAAGACAT	(186-188)	
	Calyptratae	Cal-gen-S263	AAAATAACAATACAGGACTCATATTA	238	
		Cal-gen-A264	TAATACGCTTACATACATAAGGTATA	(236-240)	
flying insects FLY-2	Syrphidae	Syr-gen-S269	ATTAGGCTAAAACCAAGCGATTT	86	Sint et al. 2014
		Syr-gen-A270	TCGGTACAAGACCATACGATCG		
	Hymenoptera	Hym-gen-S273	CGATGTTGGTTCACCGCTC	114	
		Hym-gen-A274	CRATGAAGAGCACCGCGAT	(101-200)	
	Lepidoptera	Lep-gen-S274	GCAAGCCGTATTAAGGCGAT	134	
		Lep-gen-A275	CCCATCGCTGGTCAGAGTTC	(133-135)	
	Bibionidae	Bib-gen-S271	TTCCGCACAGGCAATACCTT	153	
		Bib-gen-A271	CAATAAAGAGAACTGCTATGGGCT		
	Chironomidae	Chi-gen-S272	CCGTCAAAAGTTTCTTGTGCAG	179	
		Chi-gen-A272	CGTAGCAACCATGGTAGTCCTCT	(175-188)	
	Cinara sp.	Cin-sp-S275	TCTGGGCGGTGTCGGAC	251	
		Cin-sp-A277	GCACAGCAAGATTGGAGTAGG		

MOLECULAR ECOLOGY

Sint D, Raso L, Traugott M (2012) Advances in multiplex PCR: balancing primer efficiencies and improving detection success. Methods in Ecology and Evolution 3, 898-905.

Sint D, Niederklapfer B, Kaufmann R, Traugott M (2014) Group-specific multiplex PCR detection systems for the identification of flying insect prey. PLoS ONE 9, e115501.

Sint D, Thurner I, Kaufmann R, Traugott M (2015) Sparing spiders: faeces as a non-invasive source of DNA. Frontiers in Zoology 12.

S5: False negative samples

Table S5: Overview of false-negative samples (regurgitates from carabid beetles) that were excluded from the analysis.

Valley	Pioneer stage	*Nebria germari*	*Nebria jockischii*	*Nebria rufescens*	*Oreonebria castanea*	Σ
Gaisbergtal	early	1	1	0	0	**2**
Gaisbergtal	late	0	1	0	3	**4**
Rotmoostal	early	1	4	0	0	**5**
Rotmoostal	late	3	4	2	4	**13**
Langtal	early	5	1	0	0	**6**
Langtal	late	0	0	0	0	**0**
Σ		**10**	**11**	**2**	**7**	**30**

S6: Prey detection rates for the predators from 3 valleys and 2 pioneer stages

Data table ***Supplementary_Data_S6.xlsx***:

Proportion of predators (%) testing positive for each prey broken down by predator, valley, area (1832 predator individuals). Values for cannibalism are missing due to the inability to measure within-species predation with molecular methods.

MOLECULAR ECOLOGY

S7: Community composition of predators and prey

Data table ***Supplementary_Data_S7.xlsx***:

Community composition data of predators and prey. Predator data is given as average catch per live pitfall trap. Data of flying insects and collembolans is reported as total catch over the 2 weeks sampling period with Malaise trap and grey bowl (flying insects) and permanent pitfall traps (collembolans) respectively.

Assessment of the predator community:
Catch data from the pitfall grids described in the main article was used to describe the predator community.

Assessment of the prey community:
Flying insect prey was assessed by pooled catch totals from one Malaise trap and one grey bowl (23x23 cm) operated from 12th to 23rd July 2010 at each of the six sites. A saturated salt solution with a drop of odourless detergent was used as preservation liquid and all traps were emptied daily. Dipterans were determined to families, other insects are given as orders, except for the aphid *Cinara* sp.. Please note that numbers of Cinara sp. available to the predators are likely underestimated in the 'flying insect data' as these aphids are no regular member of the glacier foreland community but inhabit pine forests. The aphids were blown by the wind to the glacier forelands and provide thus a classical example of random Aeolian dropout. This means that when they were present, they were found in high numbers more or less immobilized on the ground. Collembola data stem from sets of each 5 permanent pitfall traps (50% ethylene glycol) surrounding the pitfall grids used for live catches. Permanent pitfall traps were emptied once at the end of the 2 weeks period. Collembolans were not determined further, but catches were dominated by surface-active Entomobryiidae.

Predator and prey communities from the six sites were compared by principal components analysis (PCA) using correlations of square root transformed catch counts in Canoco 5 (ter Braak & Šmilauer 2012).

Results of predator and prey community analysis (Figure S7):
For the predators, axis 1 separated Langtal from the other two valleys, mostly due to differences in the two Pardosa species and *Nebria germari*, while the much weaker axis 2 showed changes from the early to the late pioneer stage mostly caused by the near absence of *N. rufescens* in the early stage.
Among the prey the differences between early and late pioneer stage were more important than valley differences and appeared on axis 1. Nearly all flying insect groups increased in the late pioneer stage, only the collembolans declined markedly. Valley differences, mostly of Gaisbergtal, were associated with a few groups only, such as the sporadically, but then massively occurring aphid *Cinara* sp., flying beetles, and three brachyceran families.

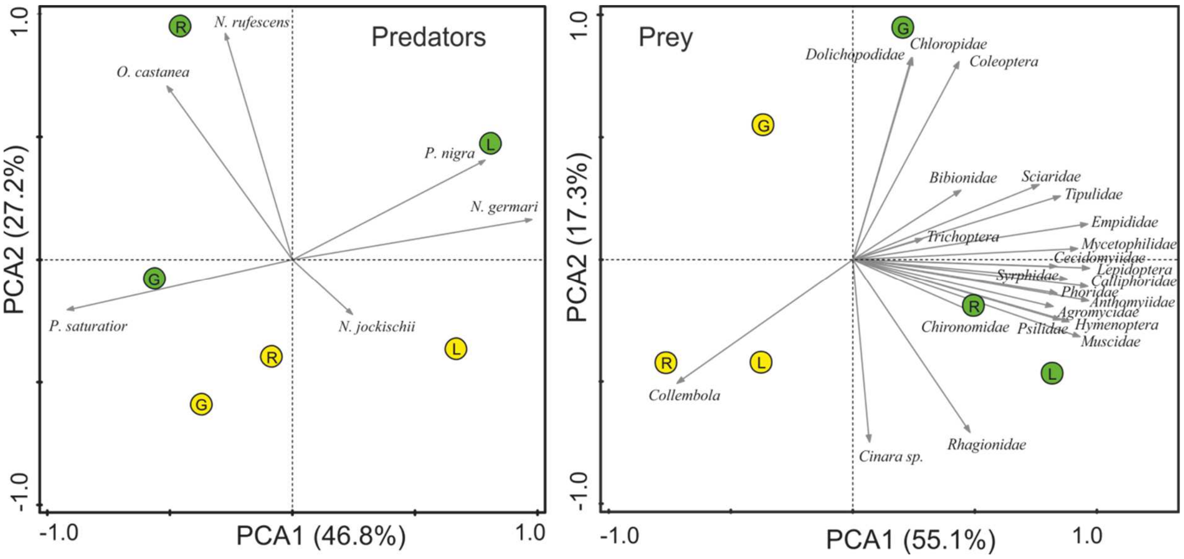

Figure S7: Principal components analysis (PCA) of predator and prey communities from the early (yellow) and late (green) pioneer stages of the three valleys Gaisbergtal (G), Rotmoostal (R), and Langtal (L). Fractions of explained variance are given with the axis labels.

Hodkinson *et al.* (2001) investigated the densities of chironomids in an Arctic glacier foreland by placing a series of water filled traps at the ground. To enable a comparison between those densities and the ones present in Alpine glacier forelands (this study), we considered here only catches from the grey bowls but not from the malaise traps. Numbers from both studies were normalized on caught chironomids per day and 500 cm² water surface. As no precise numbers are stated in Hodkinson et al. (2001), they were estimated from the presented figures.

Table S7: Number of chironomids per day and 500 cm² surface area, collected in one Arctic and three Alpine glacier forelands on early and late pioneer sites.

Region	Glacier foreland	Time since deglaciation	Chiron./day	Study
Arctic	Midtre Lovénbreen	2 years	2.65	Hodkinson et al. 2001
Alpine	Gaisbergtal	0-8 years	0.36	this study
Alpine	Rotmoostal	0-8 years	0.58	this study
Alpine	Langtal	0-8 years	0.44	this study
Arctic	Midtre Lovénbreen	16 years	132.63	Hodkinson et al. 2001
Alpine	Gaisbergtal	13-20 years	0.29	this study
Alpine	Rotmoostal	13-20 years	1.67	this study
Alpine	Langtal	13-20 years	5.02	this study

MOLECULAR ECOLOGY

Hodkinson ID, Coulson SJ, Harrison J (2001) What a wonderful web they weave: spiders, nutrient capture and early ecosystem development in the high Arctic - some counter-intuitive ideas on cummunity assembly. Oikos 95, 349-352.

ter Braak CJF, Šmilauer P (2012) Canoco reference manual and user's guide: software for ordination (version 5.0). Ithaca, NY, USA, Microcomputer Power

S8: Valley comparison of prey spectrum across all predators

Prey detections from all predators and both the early and the late pioneer stages were pooled to summarize differences between the three valleys. The basic pattern of prey usage was very similar with differences only in details. Gaisbergtal differed from the other two valleys by higher predation on *Pardosa saturatior* (which was abundant only there), Tipulidae, calyptrate flies (mostly Muscidae and Anthomyiidae), and less predation on the aphid *Cinara* sp. which also rarely occurred there. Otherwise there was only a comparatively low predation on Linyphiidae in Rotmoostal (Figure S8).

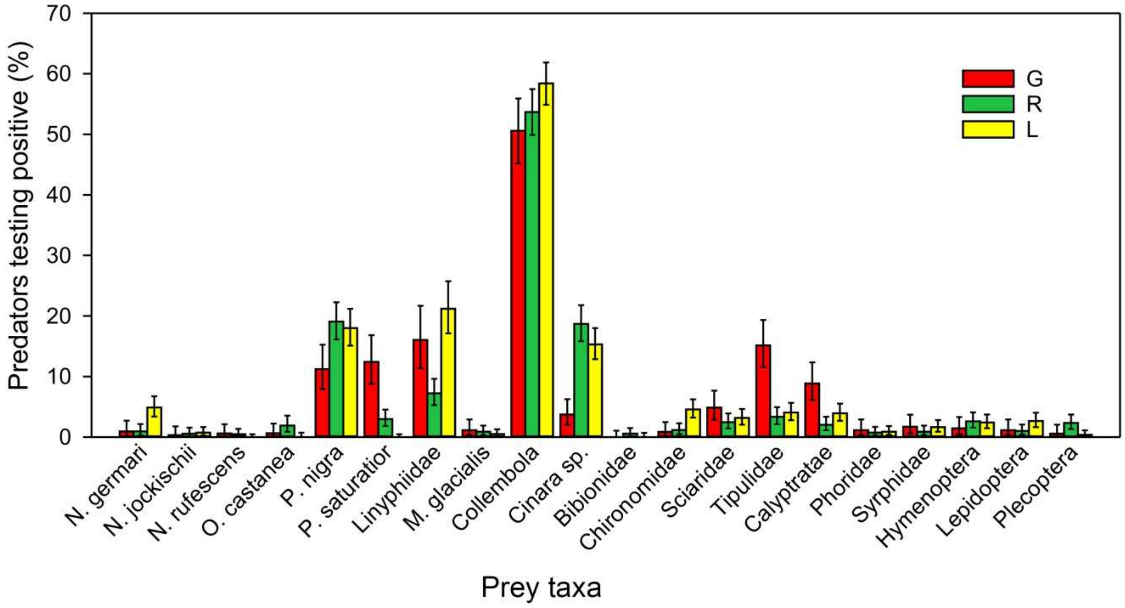

Figure S8: Prey usage differences between the three valleys Gaisbergtal (red), Rotmoostal (green), and Langtal (yellow). Pooled data from both pioneer stages and all predators. Error bars are binomial confidence intervals (95%) calculated with an Excel Add-In by John C. Pezzullo (http://statpages.org/confint.html) which follows Clopper & Pearson (1934).

Clopper, CJ & Pearson, ES (1934) The use of confidence or fiducial limits illustrated in the case of the binomial. Biometrika 26, 404-413.
